# Identification and Analysis of Cuticular Wax Biosynthesis Related Genes in *Salicornia europaea* Under NaCl Treatment

**DOI:** 10.3390/ijms26062632

**Published:** 2025-03-14

**Authors:** Richard John Tiika, Hongshan Yang, Guangxin Cui, Yanjun Ma, Solomon Boamah, Yi Li, Huirong Duan

**Affiliations:** 1 College of Forestry, Gansu Agricultural University, Lanzhou 730070, China; tiikarichard@hotmail.com (R.J.T.); mayanjun@gsau.edu.cn (Y.M.); 2 Lanzhou Institute of Husbandry and Pharmaceutical Science, Chinese Academy of Agricultural Sciences, Lanzhou 730050, China; yanghongshan@caas.cn (H.Y.); cuiguangxin@caas.cn (G.C.); 3 College of Plant Protection, Gansu Agricultural University, Lanzhou 730070, China; solomon4408boamah@gmail.com

**Keywords:** *Salicornia europaea*, cuticular wax, NaCl treatment, gene expression, wax biosynthesis

## Abstract

Salinity is a major environmental factor that adversely affects plant growth and production. Cuticular wax protects plants against external environmental stress. The relationship between cuticular wax biosynthesis and salt tolerance remains unclear in *Salicornia europaea*. This study examined the cuticle thickness, wax load, morphology, composition, and the expression of cuticular wax biosynthesis gene identification and expression. The results showed that 600 mM NaCl treatment enhanced the cuticle thickness and total wax load; crystal wax structures were also observed after NaCl treatment. The cuticular wax was mainly composed of fatty acids, alcohols, alkenes, and esters. The alcohol class accounted for the largest proportion, with docosanol (C_25_H_54_OSi) being the main specific alcohol compound, followed by fatty acids and alkanes. After a sequence database search, six fatty acyl-CoA reductases (FARs), sixteen wax synthase/diacylglycerol acyltransferases (WS/DGATs), three fatty alcohol oxidases (FAOs), five eceriferums (CERs), and eight mid-chain alkanes (MAHs) were identified as the putative wax biosynthesis enzymes. Their expression analysis revealed a differential response to 100 and 600 mM NaCl treatment and reached the highest level at 12 h or 48 h. The genes that were evidently upregulated with higher fold changes under salinity, such as *SeFAR1*, *SeFAR2*, and *SeFAR3* are implied to synthesize primary alcohols, and *SeWSs* convert the primary alcohols to wax esters; *SeCER1* and *SeCER3* are also supposed to catalyze the conversion of aldehydes to alkanes while *SeMAH7* catalyze alkanes to secondary alcohols in *S. europaea* in response to NaCl treatment. This study demonstrated that both the decarbonylation and acyl-reduction wax biosynthesis pathways may not be independent from each other.

## 1. Introduction

Abiotic stress, such as salinity, is one of the major limiting factors affecting plant growth and development and frequently result in reduced crop productivity worldwide [1]. Salinity affects about 11% of global irrigated lands and could lead to above 50% yield loss in salt-sensitive crop species including all important glycophytic crops [2]. In order to survive under salinity stress, plants have evolved comprehensive physiological, morphological, and genetic mechanisms to mitigate abiotic stress conditions [3,4]. Among the morphological mechanisms, cuticular wax thickening and cutinization of the aerial surfaces provide an essential barrier to protect plants from biotic and abiotic stress [5]. As a major component of the cuticle, cuticular wax is the outermost hydrophobic layer, serving as a barrier to restrain uncontrolled non-stomatal plant gas exchange. It also protects plant tissue from desiccation, extreme temperatures, increased UV radiation, pathogen infections, and mechanical damage, and greatly contributes to plant adaptation to salinity stress [6,7].

It is well-known that plant cuticular waxes are organic solvent-extractable complex mixtures of hydrophobic lipids, consisting mostly of very long-chain saturated fatty acids (VLCFAs) and derivatives. These VLCFAs include fatty acids (with a chain length of C_20_–C_24_), primary alcohols (C_22_–C_40_), alkanes (C_21_–C_35_), aldehydes (C_24_–C_36_), ketones (C_21_–C_35_), and diketones (C_22_–C_36_). There are different structures and compositions of cuticular waxes in different tissues and organs. Many environmental factors also considerably influence the wax composition in the same species [8]. Previous studies of *Solanum lycopersicum* epidermal wax revealed that the total cuticular wax on the stem had a higher content compared with that of the leaf [9]. Tissue wax analysis of *Thellungiella salsuginea* suggested that alkanes, ketones, and secondary alcohols (2-alcohols) were the main components in the cuticular wax of the leaves and stems, while alkanes and acids dominated in flowers [10]. Cuticular wax is synthesized on the outer membrane in the plastid of epidermal cells with the de novo C_16_ and C_18_ fatty acid synthesis. The C_16_ and C_18_ fatty acids serve as central intermediates for wax classes, and they are elongated to wax precursors of VLCFAs with C_26_ to C_34_ chains by a repeating reaction process via the fatty acid elongase (FAE) complex. Following elongation, wax components are finally generated by converting long-chain fatty acyl-CoAs via two pathways. The acyl-reduction pathway mediated by proteins encoded by *CER4* and *WSD1* generates primary alcohols and wax esters. As major wax components found in a wide range of plant species, the biosynthesis of primary alcohols is completed by the acyl-reduction pathway, in which fatty acyl-CoA reductase (FAR) converts fatty acyl-CoAs into primary alcohols. The decarbonylation pathway, involving the gene products of *CER3*, *CER1*, and *MAH1*, produces alkanes, aldehydes, secondary alcohols, and ketones [6]. The identification of these wax-related genes helps understand the production of cuticular wax and its functions.

*Salicornia europaea* is an annual succulent halophyte belonging to the *Amaranthaceae* family with extremely reduced leaves that are fairly richly branched, achieves a length of up to 35 cm, and grows luxuriantly under salt marshes (both inland and coastal) [11,12,13]. It is a potential alternative crop for saline water agriculture and the reclamation of saline lands [14]. As one of the highest salt-resistant plant species globally, the growth of this species can be promoted by 50 to 400 mM NaCl and can survive up to 1000 mM NaCl [15,16]. Thus, *S. europaea* is an exceedingly valuable model plant for researching mechanisms of salt tolerance [17,18]. Recently, several studies have identified wax biosynthesis-related genes in many plant species, for instance, in *A. thaliana* and *Oryza sativa*, 8 *FARs* were identified [19,20], 13 and 11 *WSDs* were identified in *A. thaliana* and cotton, respectively [21,22]. Moreover, 29 *CERs* were discovered in *Ziziphus jujube*, 34 *CERs* in *Passiflora edulis*, and 26 *CERs* in *S. lycopersicum* [23,24,25]. It is worth noting that, although some relevant evidence was obtained from several species, knowledge of wax related genes in *S. europaea* remains unclear. Therefore, the objective of this study was to provide a comprehensive insight into cuticular wax accumulation as well as the compounds and identify genes that are likely to be involved in the biosynthesis of wax in *S. europaea* under saline conditions.

To elucidate the relationship between cuticular wax biosynthesis and salt tolerance, we measured changes in the composition and accumulation of cuticular wax in *S. europaea* under NaCl treatment, and the expression patterns of key genes related to the cuticular wax biosynthesis were also identified. This study provides important gene resources for molecular breeding against salt stress.

## 2. Results

### 2.1. Shoot Cuticle Thickness Response to NaCl Treatment

**Figure 1:**
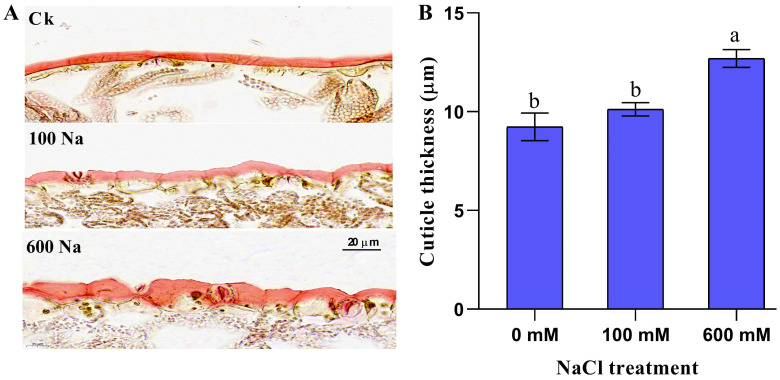
*S. europaea* under NaCl treatment after 10 d. (**A**) Shoot cuticle stained with oil red and (**B**) Cuticle thickness. Ck indicates the control, 100 Na indicates 100 mM NaCl, and 600 Na indicates 600 mM NaCl. Values are the means ± SD, and the bars with different letters indicate significant differences at *p* < 0.05 (Duncan’s test). Scale bar = 20 μm.

To evaluate potential changes in *S. europaea* cuticle response to NaCl treatment, we used light microscopy to visualize the oil red stained cuticle, and the thickness was measured (Figure 1A). The application of NaCl to *S. europaea* significantly promoted the cuticle thickness (*p* < 0.05) with increasing NaCl treatment; 600 mM NaCl had the highest cuticle thickness compared to Ck, which was 37.5% increased (Figure 1B).

### 2.2. Cuticular Wax Morphology Under NaCl Treatment

**Figure 2:**
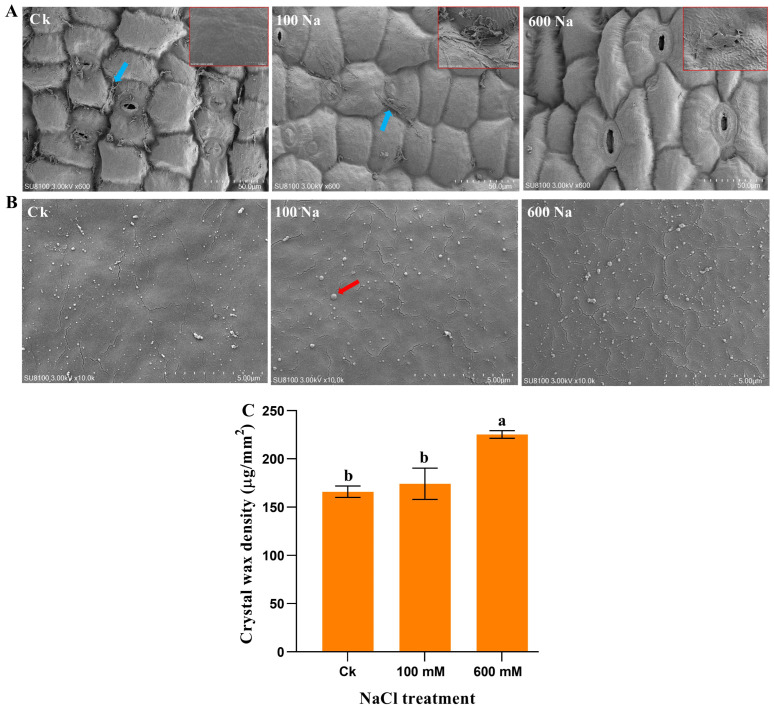
SEM images of the surface wax morphology of the *S. europaea* shoot. (**A**) Wax loss, (**B**) Wax crystal, and (**C**) Wax crystal density under NaCl treatment. The blue arrow indicates the dropping wax, red shows the crystal wax. Ck indicates the control (0 mM NaCl), 100 Na indicates 100 mM NaCl, and 600 Na indicates 600 mM NaCl. Values are the means ± SD, and bars with different letters indicate significant differences at *p* < 0.05 (Duncan’s test). Scale bar = 50 μm and 5 μm.

In this section, we examine the effects of NaCl treatment on the morphology of cuticular wax in *S. europaea*. Using Scanning Electron Microscopy (SEM), we analyzed changes in the morphology of wax under varying NaCl concentrations. Visual inspection of the SEM results revealed that wax on the shoot surface without NaCl treatment easily dropped with the smooth epidermal cell surface when the resolution was increased. The wax of the 100 mM treated shoot also dropped, but appeared better than that in the Ck. A clear wax sheath stacked firmly to the shoot surface after the 600 mM treatment was observed, especially when the resolution was adjusted (Figure 2A). Wax crystal deposition was observed on the epidermis and the density on each surface was counted (Figure 2B). The treatment of NaCl enhanced the density of wax crystals, and 600 mM had a higher density of wax crystal deposition with a 1.36-fold increase compared to Ck (Figure 2C).

### 2.3. Wax Content and GC-MS Analysis of the Wax Composition in S. europaea

**Figure 3:**
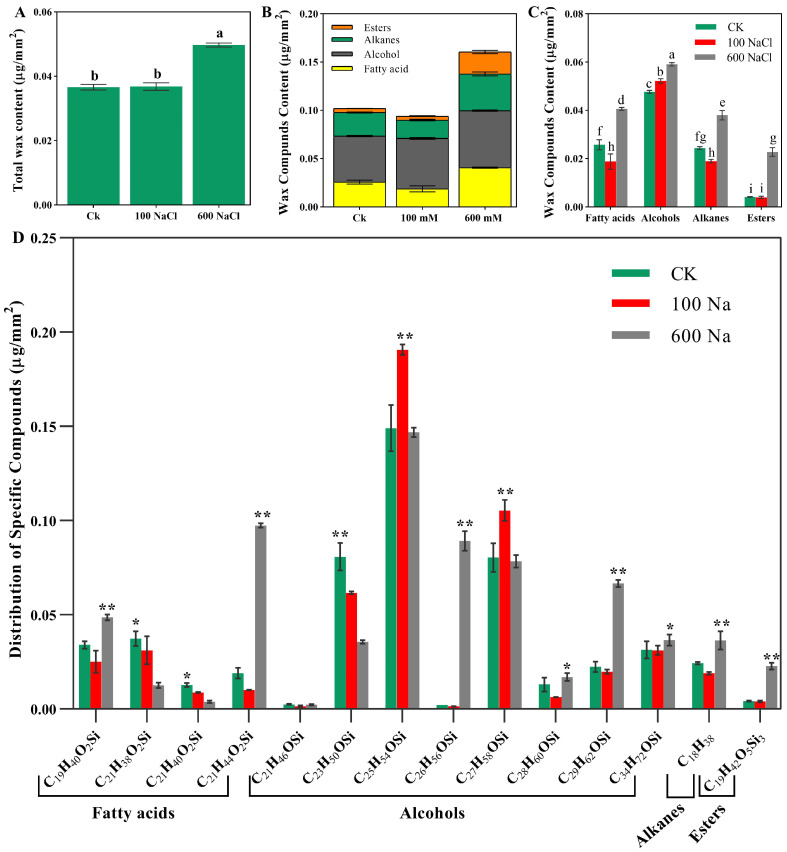
NaCl-treatment on the *S. europaea* wax. (**A**) Total wax content, (**B**) Wax compounds distribution, (**C**) Wax compound content under NaCl treatment; values are the means ± SD and bars with different letters indicate significant differences among the NaCl treatments and wax compounds at *p* < 0.05 (Duncan’s test), (**D**) Specific wax compounds; C_19_H_40_O_2_Si (palmitic acid trimethylsilyl), C_21_H_38_O_2_Si (α-linolenic acid), C_21_H_40_O_2_Si (linoleic acid), C_21_H_44_O_2_Si (stearic acid), C_21_H_46_OSi (octadecanol), C_23_H_50_OSi (eicosanol), C_25_H_54_OSi (docosanol), C_26_H_56_OSi (tricosanol), C_27_H_58_OSi (1-tetracosanol), C_28_H_60_OSi (pentacosan-1-ol), C_29_H_62_OSi (1-hexacosanol), C_34_H_72_OSi (1-octacosanol), C_18_H_38_ (n-octadecane), and C_19_H_42_O_5_Si_3_ (decanedioic acid). Ck indicates the control, 100 Na indicates 100 mM NaCl, 600 Na indicates 600 mM NaCl, single asterisk indicates significance (*p* < 0.05), and a double asterisk indicates evidently significant (*p* < 0.01).

The total wax content was quantified in *S. europaea* under varying concentrations of NaCl to evaluate how salinity stress influenced the wax composition. The results demonstrated that there was no significant difference in the total wax content between the Ck and 100 mM treatment but was significantly higher in the 600 mM treatment compared with the Ck with a 35.8% increase (Figure 3A). The cuticle wax of *S. europaea* was mainly composed of fatty acids, alcohols, alkenes, and esters. In the Ck, 25.3% fatty acids, 46.8% alcohols, 23.9% alkenes, and 4.1% esters were accumulated; 100 mM accumulated 20.0% fatty acids, 55.6% alcohols, 20.2% alkenes, and 4.2% esters; and finally, 600 mM accrued 25.3% fatty acids, 36.8% alcohols, 23.7% alkenes, and 14.2% esters (Figure 3B). Among the NaCl treatments, 600 mM significantly (*p* < 0.05) promoted all compounds with 0.58-fold, 0.24-fold, 0.56-fold, and 4.5-fold compared with the Ck (Figure 3C).

The fatty acids ranged from C_19_ to C_21_ carbon atoms in length, and the main specific fatty acid compounds or monomers were palmitic acid (C_19_H_40_O_2_Si), α-linolenic acid (C_21_H_38_O_2_Si), linoleic acid (C_21_H_40_O_2_Si), and stearic acid (C_21_H_44_O_2_Si). After NaCl treatment, C_19_H_40_O_2_Si and C_21_H_44_O_2_Si were significantly (*p* < 0.05) promoted by 600 mM NaCl while C_21_H_38_O_2_Si and C_21_H_40_O_2_Si decreased with increasing NaCl compared to Ck. The alcohols ranged in carbon chain length from C_21_ to C_36_ and the specific compounds are octadecanol (C_21_H_46_OSi), eicosanol (C_23_H_50_OSi), docosanol (C_25_H_54_OSi), tricosanol (C_26_H_56_OSi), 1-tetracosanol (C_27_H_58_OSi), pentacosan-1-ol (C_28_H_60_OSi) 1-hexacosanol (C_29_H_62_OSi), and 1-octacosanol (C_34_H_72_OSi). When the effect of NaCl treatment was examined, C_26_H_56_OSi, C_28_H_60_OSi, C_29_H_62_OSi, and C_34_H_72_OSi were significantly promoted (*p* < 0.05) by the 600 mM NaCl treatment, while 100 mM NaCl significantly increased the content of C_25_H_54_OSi and C_27_H_58_OSi compared with Ck. Among the monomers of alcohols, C_25_H_54_OSi was the most accumulated compound under NaCl treatment. The alkanes and esters were composed of n-octadecane (C_18_H_38_) and decanedioic acid (C_19_H_42_O_5_Si_3_), respectively. The specific contents of alkane C_18_H_38_ and ester C_19_H_42_O_5_Si_3_ also increased significantly (*p* < 0.05) with the 600 mM NaCl treatment compared with the Ck (Figure 3D and Figure S1).

### 2.4. Identification of Cuticular Wax Biosynthesis Proteins in S. europaea

We conducted a sequence database search for the purpose of identifying key proteins involved in the cuticular wax biosynthesis of *S. europaea* under NaCl treatment. After the sequence database search, a total of 38 putative cuticle-associated enzymes were identified; six fatty acyl-CoA reductase (FAR) members, sixteen wax synthase/diacylglycerol acyltransferase (WS/DGAT) members, three fatty alcohol oxidase (FAO) members, five eceriferums (CERs) that encode aldehyde decarbonylase members, and eight mid-chain alkane (MAH) members. All the identified enzymes were renamed as SeFAR, SeWS, SeFAO, SeCER, and SeMAH.

The protein characteristics and subcellular localization were examined using ExPASy (https://web.expasy.org/protparam/, accessed on 20 August, 2024) and Cell-PLoc2.0 (http://www.csbio.sjtu.edu.cn/bioinf/Cell-PLoc-2/, accessed on 20 August 2024) online softwares. According to the analysis, the number of amino acids in SeFARs ranged from 488 to 606. The predicted molecular weights ranged from 55.23 to 68.79 kD, and the isoelectric points ranged from 8.35 to 8.85. The subcellular localization revealed that except for SeFAR1 and SeFAR6, which were localized in golgi apparatus, all the other members were in the chloroplast. The amino acid lengths of SeMAH ranged from 121 to 513. The predicted molecular weights ranged from 13.70 to 59.09 kD, and the isoelectric points ranged from 5.94 to 9.33. All the SeMAHs were localized in the endoplasmic reticulum. The SeCER amino acids were also from 114 to 625 in length, the predicted molecular weights ranged from 12.83 to 73.02 kD, and the isoelectric points ranged from 5.53 to 9.19. Except for SeCER5, which was localized in the chloroplast, all the other members were in the vacuole. For the SeWS amino acids, the lengths ranged from 153 to 541, the molecular weights of the predicted amino acids ranged from 16.56 to 60.68 kD, and the isoelectric points ranged from 6.39 to 9.43. The subcellular localization showed that the SeWSs were localized in the cytoplasm, cell membrane, chloroplast, nucleus, and peroxisome. Finally, the amino acids of SeFAO ranged from 729 to 813, the molecular weights ranged from 79.36 to 90.22 kD, and the isoelectric points ranged from 8.31 to 8.85. Except for SeFAO2, which was localized in the chloroplast and mitochondrion, the rest were in the chloroplast and vacuole (Table 1).

### 2.5. Multiple Sequence Alignment of Cuticular Wax Biosynthesis Proteins

**Figure 4:**
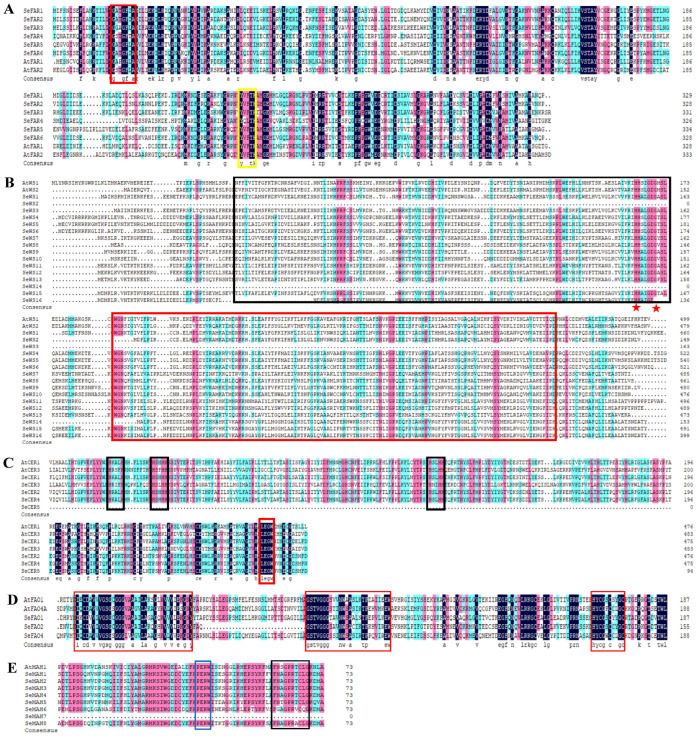
Multiple sequence alignment. (**A**) Sequence alignment of the SeFAR proteins; sequence in the red box indicates the binding site motif and the yellow box shows the active site motif. (**B**) Sequence alignment of the SeWS/DGAT proteins; the black and red boxes indicate the WES-acyltransf and DUF1298 domains, and the red stars show the position of the active site motif. (**C**) Sequence alignment of the CER proteins, His-rich motifs are indicated in the black boxes, and the red box indicates the LEGW motif. (**D**) Sequence alignment of the FAO proteins, the flavin-binding, and cytochrome *c* family heme-binding sites are indicated in the red boxes. (**E**) Sequence alignment of the MAH proteins; the PERF domain and heme-binding region are indicated with blue and black boxes, respectively.

To further characterize the wax enzymes of *S. europaea*, we carried out multiple sequence analysis using ClustalW in MEGA 5.0 software. The sequence alignment of the SeFARs of both the *A. thaliana* and *S. europaea* results contained the binding site motif G*X*_2_G*X*_2_(A) and the active site motif Y*X*_3_K (Figure 4A). WES-acyltransf and DUF1298 domains were found in the SeWS proteins in the aligned sequence, with the conserved catalytic motif HH*X*_3_DG (Figure 4B). The presence of the flavin-binding site G*X*G*X*GG*X* and cytochrome *c* family heme-binding site C*X*_2_C*X*_2_GC were used to confirm the FAO family sequence (Figure 4C). For the CER protein sequence alignment, three His-rich motifs H*X*_3_H, H*X*_2_HH, H*X*_2_HH, and the LEGW motif were discovered as the clusters that characterized the CER proteins (Figure 4D). The PERF domain and heme-binding region were used to validate the MAH protein sequence; P-E-R-W and F*X*_2_G*X*R*X*C*X*G were revealed as the PERF domain and heme-binding region, respectively (Figure 4E).

### 2.6. Phylogenetic Relationship

**Figure 5:**
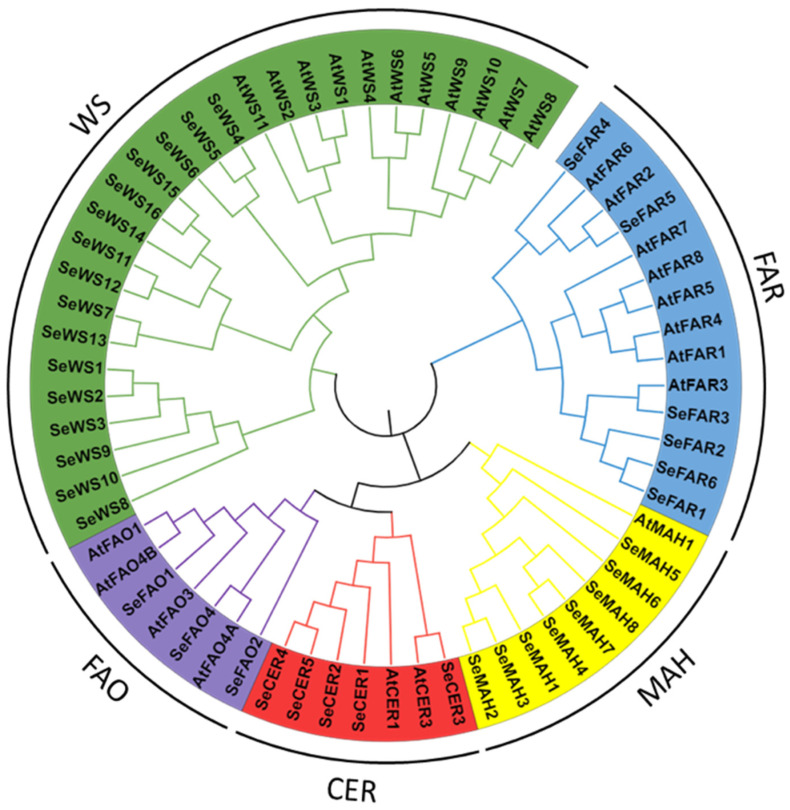
Phylogenetic tree of the *FAR*, *WS*, *FAO*, *CER*, and *MAH* genes of *A. thaliana* and *S. europaea*.

To determine if the putative proteins identified shared functional characteristics and phylogenetic relationships, a phylogenetic tree was constructed using MEGA 5.0 software. The phylogenetic tree revealed that *A. thaliana* FAR3 and *S. europaea* FAR3 shared high homology, while SeFAR5 was also a homolog of AtFAR2. WS proteins were clustered into three groups, with SeWS4, SeWS5, and SeWS6 sharing the same cluster with the AtWSs. Among the FAOs, all of the sequences shared one clade, with SeFAO4 and AtFAO4A sharing a homolog. Most SeCER proteins were clustered in one group, while *SeCER3* was clustered with *AtCER3*, indicating their similar functions. Finally, the MAH proteins were clustered in one group (Figure 5).

### 2.7. Conserved Motifs of Wax Biosynthesis Proteins in S. europaea

**Figure 6:**
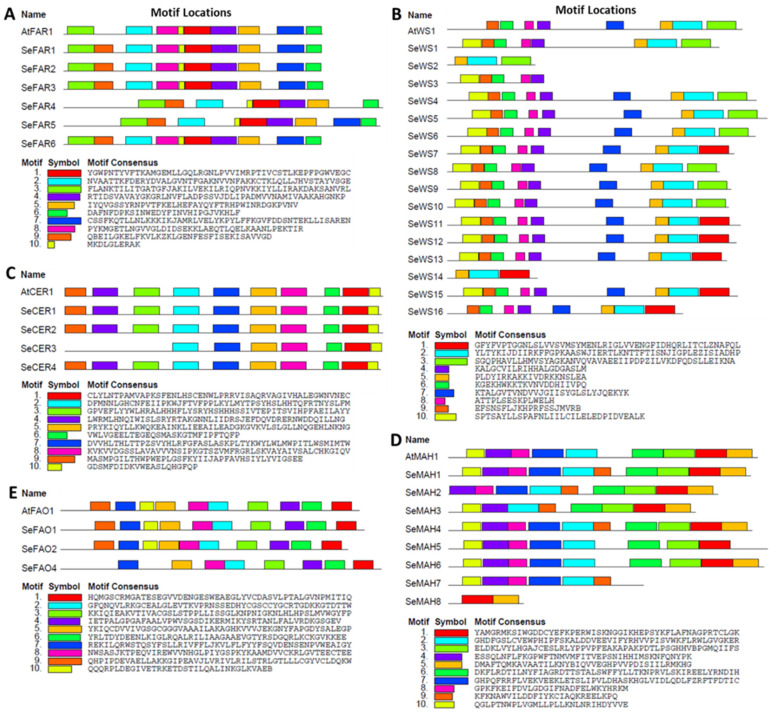
Distribution of conserved motifs. (**A**) SeFAR conserved motifs; (**B**) SeWS conserved motifs, (**C**) SeCER conserved motifs, (**D**) SeMAH conserved motifs, and (**E**) SeFAO conserved motifs. The different colored boxes represent different motifs and their position in each wax biosynthesis protein. Motifs are indicated by a colored box in the legend at the bottom (1–10).

To understand the structure and functional characteristics of the key wax biosynthesis proteins, we identified conserved domains and motifs in the proteins of SeFARs, SeWSs, SeCERs, SeMAHs, and SeFAOs using the MEME search tool. The conserved motifs of SeFARs had high similarities, except for motif 8, which was missing in SeFAR4 and SeFAR5. Among the motifs, motif 3 contained the conserved binding site motif, and the active site motif was contained in motif 1 (Figure 6A). The conserved motifs of the SeWS proteins were either eight or nine in number, except for SeWS2, SeWS3, and SeWS14, which had three, five, and three motifs, respectively. The active site motif sequences were in motif 4 (Figure 6B). In the SeCER conserved motif distributions, except for SeCER3, all of the other members had all 10 motifs. The three His-rich motifs and the LEGW motif were also present in motif 2, motif 3, and motif 1, respectively (Figure 6C). Among the SeMAH members, only SeMAH1 and SeMAH4 contained all of the 10 conserved motifs, and both the PERF domain and heme-binding region sequence were conserved in motif 1 (Figure 6D). SeFAO4 contained only eight conserved motifs, but the other members contained all ten motifs. The flavin-binding site sequences were present in motif 5, while the cytochrome *c* family heme-binding site sequences were conserved in motif 2 (Figure 6E).

### 2.8. Expression of Wax Biosynthesis Genes Under NaCl Treatment

**Figure 7:**
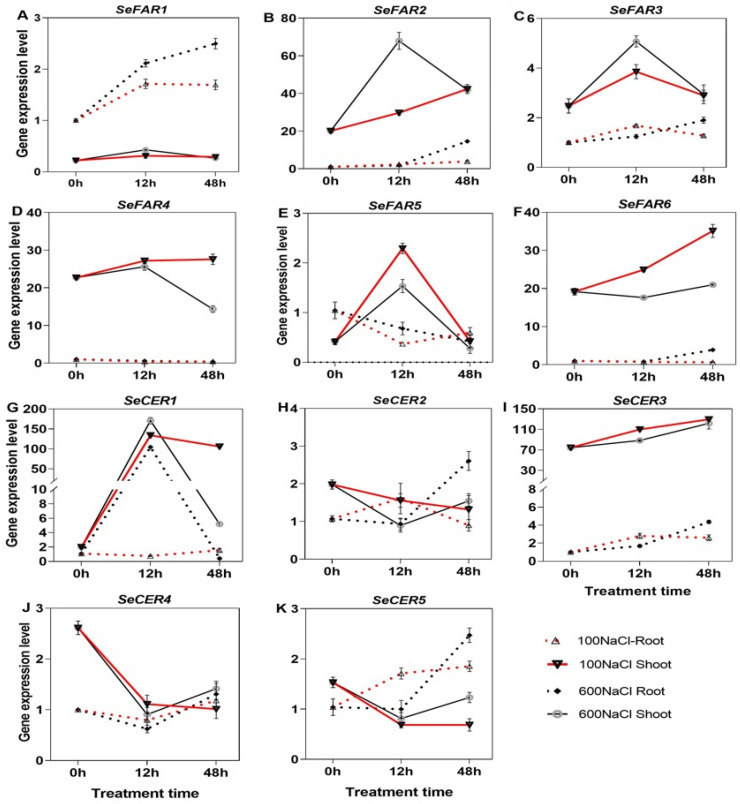
qRT-PCR expression of the *SeFAR* and *SeCER* genes in *S. europaea*. (**A**–**F**) *SeFARs* and (**G**–**K**) *SeCER* genes. Values are the means ± SD and the bars indicate error bars.

To investigate the expression pattern of the *S. europaea* wax biosynthesis genes (*SeFAR*, *SeWS*, *SeMAH*, *SeFAO*, and *SeCER*), we performed qRT-PCR analysis of the roots and shoots using specific primers. Several genes showed variations that were more extensive in expression during the different exposure periods as well as among the different tissues. The expression of *SeFARs* showed varied differential profiles under 100 and 600 mM at the 12 and 48 h periods in the shoot and root (Figure 7A–F). For instance, *SeFAR1* was significantly expressed in the root and much higher under 600 mM NaCl at 48 h, while *SeFAR2* and *−3* were significantly expressed in shoot under 600 mM NaCl at 12 h. In addition, *SeFAR2*, and *−6* in the shoot under 100 mM NaCl were increasingly expressed and peaked at 48 h, with fold increase of 1.1, and 0.8, respectively compared with the Ck (Figure 7B,D,F); the expression profiles of *SeFAR5* was higher, with a 4.5-fold increase under 100 mM for 12 h of treatment compared with the Ck (Figure 7E). In the case of *SeCER*, the expression showed that *SeCER1* was significantly upregulated in the shoot with 66.7-fold and 85.2-fold higher under 100 mM NaCl and 600 mM NaCl at 12 h, respectively. Also, the *SeCER1* in root was expressed remarkably under 600 mM NaCl at 12 h with 96.7-fold increase compared with the Ck (Figure 7G). The increased expression of *SeCER3* was significantly higher in the shoot than root, however, no significant difference was observed among the treatment times (Figure 7I). Similar expression patterns were observed between *SeCER2* and *SeCER5* (Figure 7H–K).

**Figure 8:**
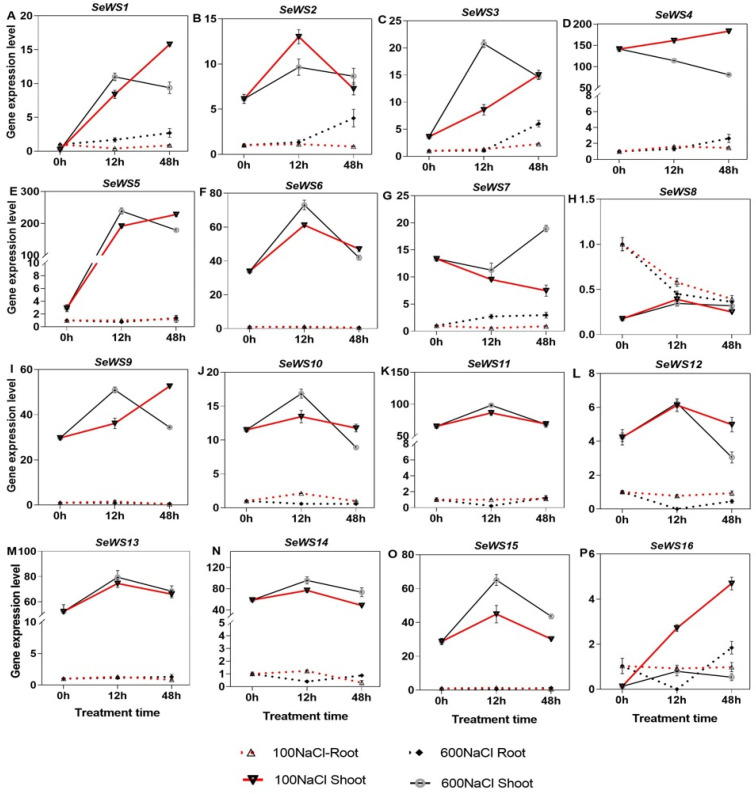
qRT-PCR expression of the *SeWS* genes in *S. europaea*. (**A**–**P**) *SeWS1* to *SeWS16*. Values are the means ± SD and the bars indicate error bars.

The expression profiles of 15 *WS* genes were significantly higher and one member was lower in the shoot than root under both NaCl contents (Figure 8A–P). The expression of *SeWS2*, *−6*, *−10*, *−11*, *−12*, *−13*, *−14*, and *−15* in shoot was significantly enhanced by both NaCl concentrations at 12 h but decreased at 48 h. Moreover, the expression of *SeWS1*, *−3*, *−5*, *−9*, and *−16* significantly increased then peaked under 100 mM NaCl at 48 h in the shoot; also, *SeWS1*, *−3*, and *−5* recorded significant fold increase of 48.3, 4.7, and 80.5 under 600 mM NaCl at 12 h in the shoot compared with the Ck. Even though *SeWS8* was higher in the root than shoot, it was downregulated at 48 h under both NaCl contents (Figure 8H,P).

**Figure 9:**
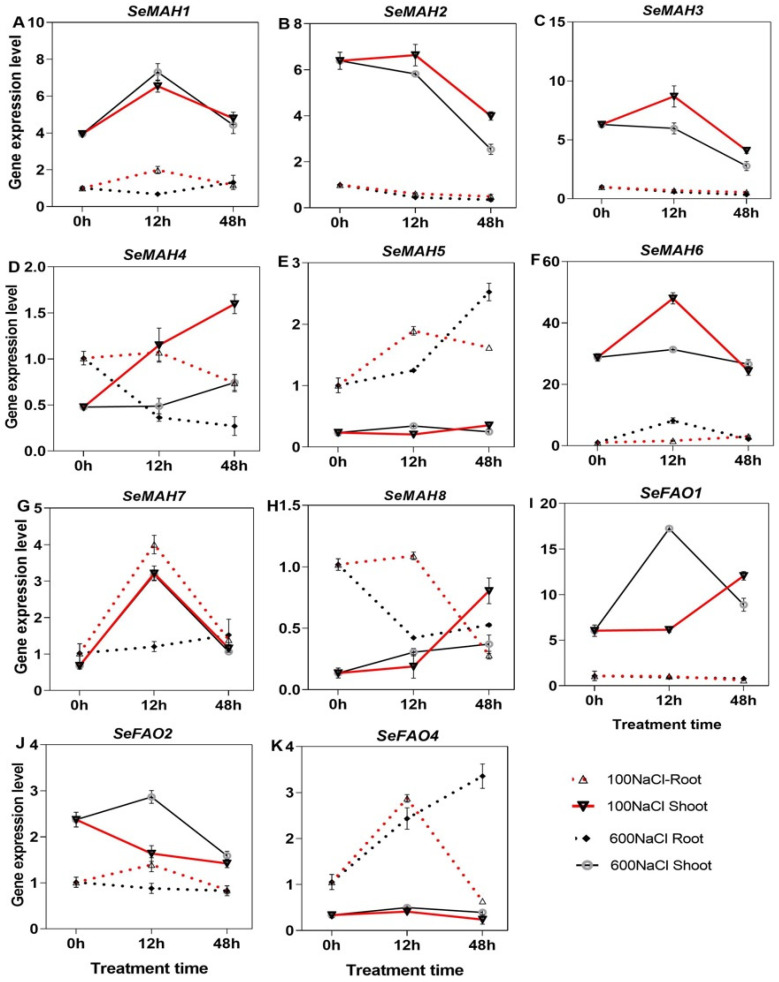
qRT-PCR expression of the *SeMAH* and *SeFAO* genes in *S. europaea*. (**A**–**H**) *SeMAHs* and (**I**–**K**) *SeFAOs*. Values are the means ± SD and the bars indicate error bars.

The expression of *SeMAH1*, *−2*, *−3*, and *−6* genes was evidently higher under both NaCl treatment in the shoot than root, for *SeMAH5*, the expression was more higher in the root than shoot (Figure 9A–C,F). The expression of *SeMAH1*, *−2*, *−3*, *−6*, and *−7* in the shoot peaked highly at 12 h but dropped at 48 h of treatment period under100 mM NaCl concentration. *SeMAH7* gene had the highest fold increase of 3.7 under 100 mM NaCl and 3.6 under 600 mM NaCl for 12 h in shoot compared with the Ck. The expression of *SeMAH4* was upregulated under both NaCl contents at 48 h in the shoot. Among the *SeFAO* genes, *SeFAO1* and *−2* were more expressed in the shoot than root while *SeFAO4* was more expressed in the root than shoot (Figure 9I–K).

## 3. Discussion

The cuticle, mainly composed of wax and cutin, is the outermost natural barrier in plants, which plays an important role in preventing water loss [26]. Salt stress has been shown to induce cuticle thickness and the accumulation of epidermal wax in plants [27,28,29]. This study suggested that the *S. europaea* shoot changed its cuticle thickness to enhance salt tolerance It is suggested that reduced epidermal permeability and enhanced water retention are perhaps achieved by increasing the total wax accumulation at a low concentration of salt stress. Nevertheless, our study concurrently enhanced the total wax load and content of wax compounds under high NaCl content, which might have reduced the epidermal permeability and increased the water use efficiency to resist NaCl stress. We also observed crystal wax structures in *S. europaea*, and the typology of wax was aligned with that proposed by Tomaszewski and Zieliński [30]. High NaCl treatment was suggested to enhance the density of the shoot surface crystal wax structures.

Wax constituents differ in various plant species; the cuticular wax of the *A. thaliana* stem is composed of a mixture of alkanes and ketones, with alcohols, aldehydes, and fatty acids accounting for less than 25% of the total [31]. In transgenic *Medicago sativa* leaves, alcohols constituted the highest wax constituents under abiotic stress [32]. In the case of *S. europaea* under NaCl treatment, the cuticular wax was composed of four compounds: alcohols, fatty acids, alkenes, and esters; 600 mM NaCl evidently enhanced their accumulation, respectively. After NaCl treatment, alcohols accounted for the largest component of the wax (73.7%), with C_25_H_54_OSi as the main alcohol component in *S. europaea*, suggesting their critical role in forming a thicker cuticular wax to combat salinity stress, giving their ability to form hydrophobic substances [33,34]. Fatty acids and alkanes are also reported to contribute to an impermeable cuticle barrier [4,10], and they constituted the second and third largest wax compounds in *S. europaea* under NaCl treatment.

Wax biosynthesis enzyme genes mediating wax accumulation in stress response have also been reported, however, it is still important to evaluate their genetic characteristics and roles in *S. europaea* under NaCl treatment. In this study, binding site motif G(A/S)(T/A)G(F/I/T/A) and an active site motif Y(V/Q/T)K were found to be present in *SeFARs*. Previous studies showed that similar motifs of FAR reported in five model plant species and the mutation of the two glycines (G), tyrosine (Y) and lysine (K) amino acid residues play a direct role in enzymatic activity [35,36]. In line with our study, two typical domains, WES-acyltransf and DUF1298, with conserved catalytic motif HH*X*_3_DG were also discovered in WS proteins [37,38,39]. The WES-acyltrans domain was reported to catalyze the reaction of CoA-dependent acyltransferase with fatty alcohol to form a wax ester [38]. Both histidines in the HH*X*_3_DG motif were also shown to be essential for wax ester synthase/acyl-CoA:diacylglycerol acyltransferase catalysis [37]. *A. thaliana* and *Candida cloacae* (*fao1* and *fao2*) contain the consensus sequence for the cytochrome *c* family heme-binding and flavin-binding signatures, as discovered in *S. europaea* [40,41]. The three His-rich motifs (H*X_3_*H, H*X_2_*HH, and H*X_2_*HH) and the LEGW motif were contained in SeCERs that were similar to Chaudhary, et al. [42], and have been proposed for several integral membrane enzymes catalyzing desaturations and alkane hydroxylation [43].

The synthesis of plant wax consists of a series of complex biological processes in which multiple genes are involved. After identifying and conducting an expression analysis of the cuticle wax genes, the expression patterns were differentially regulated by NaCl treatment in the shoots and roots at different time points. FAR reduces VLCFA-CoAs to primary alcohols, which in turn act as intermediate metabolic end products to participate in the formation of cuticular wax [35]. The results of this study showed that the *SeFAR* genes were significantly expressed; *SeFAR2*, −*3*, and *−5* had the highest expressions in the shoot under NaCl stress. The expression of *AtFAR3/CER4* in *A. thaliana* was highly upregulated in the aerial organs and was responsible for the synthesis of primary alcohols and cuticular wax [44]. Interestingly, in this study, *SeFAR3* had a close phylogenetic relationship with *AtFAR3* and was also upregulated in the shoot under 600 mM NaCl treatment, indicating a conserved role in the synthesis of primary alcohols and cuticular wax in *S. europaea*. According to Domergue et al. [20], the expressions levels of the *AtFAR1*, *AtFAR4*, and *AtFAR5* genes were increased in the root endodermal cells under high salt stress and were confirmed as alcohol-forming FARs; a similar phenomenon was observed in *S. europaea FAR1*, whose expression was also enhanced by high NaCl stress in the root than shoot, suggesting that *SeFAR1* in the root may play a role in alcohol-formation or have other functions under NaCl treatment in *S. europaea*. The response of *SeFARs* to NaCl treatment may also contribute to the large amounts of alcohols content in in *S. europaea*. WS catalyzes the esterification of primary alcohols (produced by FARs) with acyl-CoAs to form wax esters [45] and is also a key player in the acyl reduction pathway of wax biosynthesis. The expression of 15 *SeWS* genes in *S. europaea* were upregulated in response to NaCl treatment in shoot than root; *SeWS1*, *−3*, and *−5* responded to 600 mM NaCl with fold increase of 48.3, 4.7, and 80.5 compared with the Ck. The overexpressing of *WSD1* in *A. thaliana* showed a higher deposition of epicuticular wax crystals and increased leaf and stem wax load under stress [46]. Therefore, it can be hypothesized that *S. europaea WS* genes, especially *SeWS1*, *−3*, and *−5*, might have a similar function to *AtWSD1* and play a specific role in wax ester metabolism under NaCl stress; this may be attributed to the high esters content under 600 NaCl stress in *S. europaea*.

**Figure 10:**
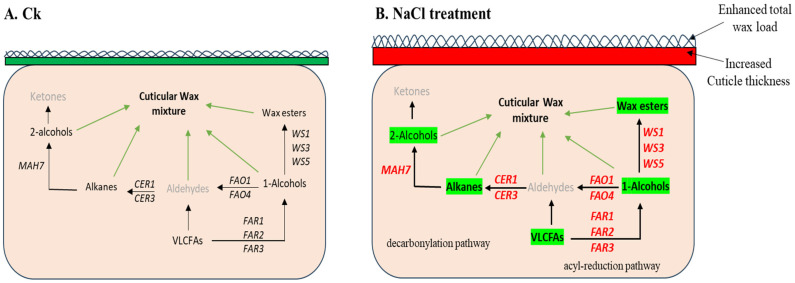
Graphical model of wax biosynthesis under NaCl treatment in *S. europaea*. (**A**) Control and (**B**) NaCl treatment. Genes highlighted in red bold indicate significant expression, green highlighted words are wax compounds promoted by the wax biosynthesis genes, black bold arrows show the expressed gene promotion of wax compounds, and green lines indicate the wax compound contribution to the cuticular wax mixture.

In the decarbonylation pathway of wax biosynthesis, CERs play core role in alkane biosynthesis. Under NaCl treatment, the *S. europaea CERs* were distinctively expressed, especially *SeCER1*, which was expressed increasingly with a fold change 66.6, 85.2, and 96.7 in the shoot and root with the different treatment times and NaCl contents. In *A. thaliana*, *AtCER1* and *AtCER3* are reported to catalyze the conversion of long-chain aldehydes to alkanes, a key step in wax biosynthesis, and thereby increase the overall wax load [6,47]. Interestingly, *AtCER3* share close a phylogenetic relationship with *SeCER3*, indicating that they may have similar functions that might contribute to the high alkane content in *S. europaea*, especially under the 600 mM NaCl treatment. Furthermore, the expression of *SlCER1* and *SlCER3* was significantly upregulated in one-month-old *S. lycopersicum* plants under stress, and the overexpression of *SlCER1-1* significantly increased very-long-chain (VLC) alkane biosynthesis and wax accumulation. MAH enzymes hydroxylate alkanes at the subterminal carbon to form secondary alcohols, for instance, *AtMAH1* converts wax alkanes to secondary alcohols and ketones [6]. The expression of *SeMAHs* was differentially expressed under the NaCl contents, however, *SeMAH7* was revealed to have 3.7and 3.6-fold increase in the shoot under 600 mM NaCl for 12 h period. This suggests that under NaCl treatment, *SeMAHs*, especially *SeMAH7*, possibly catalyze wax alkanes to secondary alcohols and ketones in *S. europaea* (Figure 10), as reported in *A. thaliana* [6]. The expression of *SeMAH5* higher in the root than shoot possibly plays a different role rather than wax alkane conversion. However, we recommend further functional analysis for *SeMAH5*, and *−7*.

FAOs have been reported to catalyze the oxidization of alcohols to produce aldehydes, the second step of the alkane oxidation pathway [48]. In *S. europaea*, *SeFAO1* and *−2* expression was evidently enhanced by high NaCl in the shoot than root while *SeFAO4* was more enhanced in the root than shoot. These results suggested that *SeFAO1* and *SeFAO4* are possibly involved in balancing primary alcohol and aldehyde accumulation and modulation. This study demonstrated that both the decarbonylation and acyl-reduction pathways may not be independent from each other (Figure 10), which was confirmed by Yang et al. [49]. However, detailed molecular functional research is needed for further exploration.

## 4. Materials and Methods

### 4.1. Plant Growth Conditions and Stress Treatments

Wild *S. europaea* seeds were surface sterilized, washed, and grown for 3 days (d) in a Petri dish layered with moist, aseptic filter paper under darkness. The plantlets were transferred to containers of sterilized sand, soaked in ½ Hoagland nutrient solution, and renewed every 3 d [50]. The plantlets were placed in a growth chamber with a day/night temperature of 25/22 °C with 65% relative humidity. The daily photoperiod was 16/8 h (light and darkness) with a light flux density of 600 μmol/m^2^·s for 4 weeks.

The 4-week-old seedlings with the same growth situation were divided into two treatment groups: the first group was used for cuticle thickness and wax composition analysis whereas the second group was used for the cuticle wax biosynthesis gene expression analysis. The first group was subjected to 0 (Ck), 100, and 600 mM of NaCl treatment with three replicates/treatment for 10 d. For the cuticle thickness and wax morphology, observation, the middle node (third node from bottom) of the shoot was collected and conserved in FAA and glutaraldehyde until used. For the wax composition analysis, the whole seedling was collected and dried until use. The second group of seedlings was exposed to 0, 100, and 600 mM NaCl treatment for 0, 12, and 48 h [51,52]. There were three seedlings/replicate and three replicates/treatment. The shoots and roots were collected separately and quickly frozen in liquid nitrogen and stored at −80 °C for subsequent gene expression analysis.

### 4.2. Scanning Electron Microscopy (SEM) Analysis

Cryogenic scanning electron microscopy (cryo-SEM) was used to view the cuticular wax crystallization patterns. Samples were adhered to the cryo-holder using carbon tape and cryo-adhesive and plunged into liquid nitrogen slush. A vacuum was pulled, and the sample was transferred to a Gatan Alto 2500 pre-chamber for cooling to about −170 °C. The samples were then sputter coated with platinum for 60–120 s at 40 mA (BAL-TEC Sputter Coater SCD 050, Scotia, NY, USA) and transferred to the microscope cryostage for imaging. Coated surfaces were imaged using a FEI Quanta 250 SEM (FEI Co., Hillsboro, OR, USA), and a voltage of 3.00 kV was maintained while imaging the samples.

### 4.3. Oil Red Staining Procedure for Cuticle Analysis

Oil Red at 0.4% (*w*/*v*) was dissolved in carbon tetrachloride. Samples were placed in a vial immersed with the oil red solution and stained for 5 min at room temperature with agitation. Samples were then rinsed with water for 5 min with agitation. The water rinse was repeated four times, as stain globules could remain on the substrate. Stained materials were mounted in glycerin on slides with coverslips and observed with light microscopy (Axio Imager M2; Zeiss, Oberkochen, Germany).

### 4.4. Determination of Cuticular Wax Composition

Shoot samples were submerged in 25 mL of chloroform at 60 °C, and then 40 μL of n-octadecane was added for standardization. Evaporated wax extracts were placed under nitrogen gas to dry, then 100 ul pyridine derivatization was added at 70 °C for 60 min and 100 ul O-bis (trimethylsilyl) trifluoroacetamide (BSTFA). These were blown dried with N_2_ gas and then dissolved with 1.5 mL chloroform. After being silylated, the wax composition was analyzed by GC-MS (SHIMADZU GC—2014) equipped with HP-5MS UI capillary column (30 m × 0.250 mm × 0.5 μm). The volume of the sample injected was 1 μL, and helium as the carrier gas was pumped with a flow rate of 1.0 mL/min. Initially, the temperature of the injection port was 290 °C. The column temperature condition was 50 °C for 5 min and then increased at 5 °C/min and kept for 20 min. For the mass spectral analysis, the spectral conditions were 70 eV ionization energy, transmission line, and the quaternal rod temperatures were 230 °C, 315 °C, and 150 °C, respectively, and the mass scan range was 33–500 *m*/*z* with a 5 scans/s scan speed under full-scan mode.

Then, the cuticle wax compounds were calculated using Sc ÷ Sp1 × Sp2/Wv × Cv × Df, where Sc is the standard concentration (μg/mL), Sp1 is the standard peak area, Sp2 is the sample peak area, Wv is the weighing volume (mm^2^), Cv is the constant volume (mL) and Df is the dilution factor.

### 4.5. Cuticular Wax Biosynthesis Protein Sequence Search

*A*. *thaliana* wax biosynthesis protein sequences were obtained from the *Arabidopsis* information resource (TAIR) database (http://www.arabidopsis.org/, accessed on 12 August 2024) and used as reference sequences. The wax biosynthesis-related enzymes of *S. europaea* was extracted from the *Salocornia* Genome website (www.salicorniadb.org, accessed on 12 August 2024) [53]. The sequences were analyzed using DNAMAN version 8 software, confirmed by an online BLAST search in the NCBI database (https://blast.ncbi.nlm.nih.gov/, accessed on 13 August 2024), and HMMER software version 3.3.2 [54] was used to authenticate the putative protein sequence with *A. thaliana* as a query with the e-value set to be less than 1 × 10^5^.

### 4.6. Analysis of Physicochemical Properties and Subcellular Localization of Proteins

The online analysis software ExPASy (https://web.expasy.org/protparam/, accessed on 20 August 2024) was used for the analysis of the *S. europaea* wax biosynthesis proteins, isoelectric point (pI), molecular weight (MW), and other physical and chemical information. Then, the protein subcellular localization prediction software Cell-PLoc2.0 (http://www.csbio.sjtu.edu.cn/bioinf/Cell-PLoc-2/, accessed on 20 August 2024) was used for the wax biosynthesis family protein sequence analysis of subcellular localization prediction.

### 4.7. Sequence Alignment, Phylogenetic, and Conservative Motif Analysis

All putative proteins were used to generate multiple protein sequence alignments by using the default settings of ClustalW in MEGA 5.0 software [55]. All of the identified *S. europaea* wax biosynthesis proteins including related members of *A*. *thaliana* were used for phylogenetic tree construction in MEGA 5.0 software [56], with the maximum likelihood method and 1000 bootstrap replications. The MEME (https://meme-suite.org/meme/tools/meme, accessed on 1 October 2024) website was used for the *S. europaea* wax biosynthesis protein conservative motif analysis.

### 4.8. RNA Extraction and RT-qPCR Analysis

The total RNA was extracted from approximately 100 mg of the frozen shoot and root tissue using the TransZol Up Plus RNA Kit, no. Lot#M31018 (TransGen Biotech Co., Ltd., Beijing, China) according to the manufacturer’s instructions. RNA quantity and quality were examined using a TGen Spectrophotometer (TianGen Biotech Co. Ltd., Beijing, China) using A260 nm/A280 nm and A260 nm/A230 nm ratios. The *Evo* M-MLV RT Kit, no. AG11705 (Accurate Biotechnology Co., Ltd., Hunan, China) was used to reverse transcribe the total RNA into cDNA and remove genomic DNA mixed in the cDNA as per the manufacturer’s protocol.

The MIQE guidelines [57] were used to perform the RT-qPCR analysis. Primers were designed based on the cDNA sequences using Primer 5 software (Table S1) and synthesized by TsingKe Biological Technology Co. Ltd., Xi’an, China. The *Ubiquitin-conjugating* (*UBC*) gene was used as a housekeeping/internal control gene [58], and the primer pair specificity was determined using qRT-PCR single peak melting curve analysis [57]. Three independent biological replicates were performed, and triplicate technical quantitative assays were performed using the Heiff^®^ qPCR SYBR^®^ Green Master Mix Kit (Yeasen Biotech Co. Ltd., Shanghai, China) according to the manufacturer’s protocol. The RT-qPCR analysis was performed using a QuantStudio™ 5 Real-Time PCR instrument (ABI, Life Technologies Holdings Pte. Ltd., Singapore). The relative expression was calculated according to the method of Livak and Schmittgen [59].

### 4.9. Data Analyses

The ImageJ software package (version 1.46r) was used to count the wax density with three SEM images as replicates per treatment. CaseViewer 2.4 software (3DHISTECH) was used to measure the cuticle thickness with a 20 µm image resolution; six sections were used as replicates. Duncan’s test in SPSS Statistics 20.0 was used for statistical analysis of all of the parameters measured.

## 5. Conclusions

High NaCl treatment enhanced the wax accumulation and cuticle thickness in *S. europaea*. Crystals wax structures were observed, and a high NaCl treatment was suggested to enhance the density of the crystal wax structures. The cuticle wax of the shoot was composed of alcohols, fatty acids, alkenes, and esters; the alcohols accounted for the largest component. After the sequence database search, six *SeFARs*, sixteen *SeWSs*, three *SeFAOs*, five *SeCERs*, and eight *SeMAHs* cuticular wax biosynthesis genes were identified in *S. europaea*. The expression analysis of these genes indicated significant differential expression patterns regulated by NaCl treatment among the different tissues, at time points of 0, 12, and 48 h. The expression of the various wax biosynthesis genes suggests that the salt stress response involves the induction of the acyl-reduction pathway and decarbonylation pathway.

## Figures and Tables

**Table 1:** Properties of the wax biosynthesis proteins and the subcellular localization prediction of *S. europaea*.

Protein Name	Gene ID	Amino Acids	Molecular Weight/kD	Theoretical pI	Instability Index (II)	Aliphatic Index	Subcellular Localization
SeFAR1	>Seu_jg19520	489	55.60	7.99	28.34	96.50	Golg
SeFAR2	>Seu_jg19521	488	55.23	8.76	22.97	106.82	Chlo
SeFAR3	>Seu_jg8291	492	56.02	8.66	35.31	97.05	Chlo
SeFAR4	>Seu_jg25353	606	68.79	8.35	37.58	100.02	Chlo
SeFAR5	>Seu_jg23269	600	66.79	8.85	37.48	85.07	Chlo
SeFAR6	>Seu_jg19519	488	55.27	8.85	25.69	98.81	Golg
SeMAH1	>Seu_jg13328	486	56.52	8.81	29.10	93.42	ER
SeMAH2	>Seu_jg24080	433	50.16	7.68	29.48	91.36	ER
SeMAH3	>Seu_jg24081	397	46.09	8.76	28.63	92.77	ER
SeMAH4	>Seu_jg24086	488	56.79	8.45	31.65	94.02	ER
SeMAH5	>Seu_jg24089	513	59.09	6.56	38.82	92.32	ER
SeMAH6	>Seu_jg17936	507	58.83	8.10	36.31	88.64	ER
SeMAH7	>Seu_jg15161	314	36.62	5.94	33.22	94.87	ER
SeMAH8	>Seu_jg15162	121	13.70	9.33	25.94	81.49	ER
SeCER1	>Seu_jg14489	622	73.02	8.93	37.99	94.77	Vac
SeCER2	>Seu_jg6667	625	71.99	8.93	32.36	101.52	Vac
SeCER3	>Seu_jg19922	623	71.52	9.19	35.87	103.56	Vac
SeCER4	>Seu_jg6668	620	71.37	9.00	32.14	100.13	Vac
SeCER5	>Seu_jg6669	114	12.83	5.53	47.02	71.84	Chlo
SeWS1	>Seu_jg12815	460	51.99	5.84	29.75	97.87	Cyto
SeWS2	>Seu_jg12816	149	16.87	5.91	28.51	110.60	Cm, Chlo, Nuc
SeWS3	>Seu_jg12818	163	18.75	5.42	38.08	84.91	Nuc
SeWS4	>Seu_jg6079	523	58.98	8.06	37.29	97.67	Chlo
SeWS5	>Seu_jg6080	541	60.68	8.36	39.12	96.38	Chlo
SeWS6	>Seu_jg6081	521	59.09	9.31	30.27	94.26	Per
SeWS7	>Seu_jg18079	485	53.90	9.10	34.61	90.19	Chlo
SeWS8	>Seu_jg20467	461	51.37	6.47	36.86	90.15	Cm, Chlo, Nuc
SeWS9	>Seu_jg20468	480	54.06	9.06	31.77	100.44	Cm, Chlo
SeWS10	>Seu_jg20469	476	53.23	8.70	37.97	94.98	Chlo
SeWS11	>Seu_jg20583	496	56.58	9.43	38.17	94.29	Chlo
SeWS12	>Seu_jg20584	489	55.68	9.28	44.96	87.48	Chlo
SeWS13	>Seu_jg21009	473	52.30	6.39	45.66	91.10	Chlo
SeWS14	>Seu_jg21132	153	16.86	8.60	35.14	98.82	Cm, Chlo
SeWS15	>Seu_jg21133	491	54.91	8.86	38.31	99.04	Chlo
SeWS16	>Seu_jg21134	399	44.85	8.72	41.61	96.74	Chlo, Cyto
SeFAO1	>Seu_jg28185	770	84.66	8.70	35.63	82.31	Chlo, Vac
SeFAO2	>Seu_jg3513	729	79.36	8.85	32.83	87.34	Chlo, Mito
SeFAO4	>Seu_jg12627	813	90.22	8.31	36.47	81.64	Chlo, Vac

Golg: Golgi apparatus, Chlo: chloroplast, ER: endoplasmic reticulum, Vac: vacuole, Per: peroxisome, Cm: cell membrane, Cyto: cytoplasm, Nuc: nucleus, Mito: mitochondrion.

## Data Availability

The data are contained within this article and Supplementary Materials.

## Supplementary Materials

The following supporting information can be downloaded at: https://www.mdpi.com/article/10.3390/ijms26062632/s1.
