# Supplementary figures and images for: Identification and Analysis of Cuticular Wax Biosynthesis Related Genes in Salicornia europaea Under NaCl Treatment
Source: Int J Mol Sci. 2025 Mar 14;26(6):2632. doi: 10.3390/ijms26062632 (PMC11942154; doi:10.3390/ijms26062632)

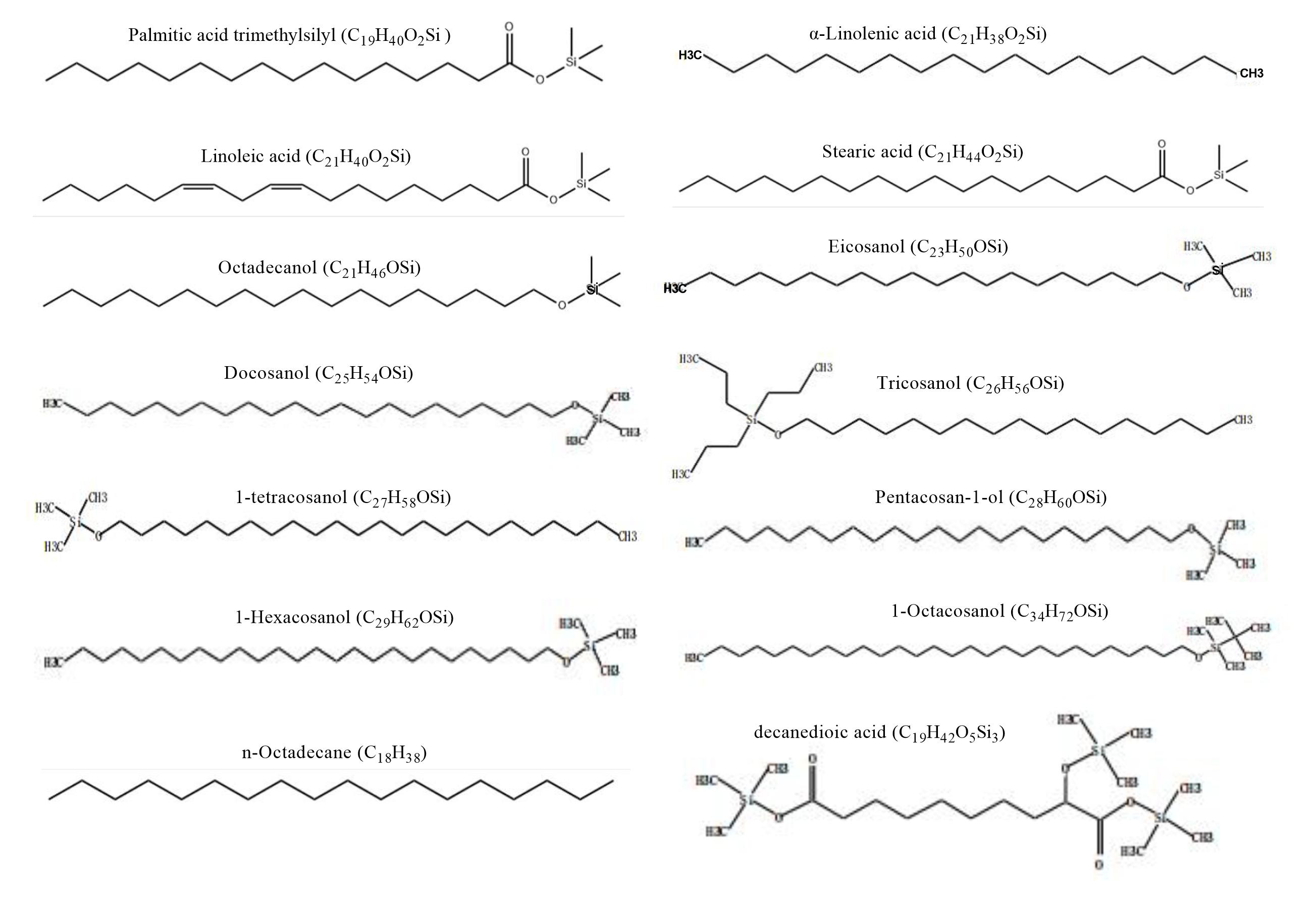

Supplement: Supplementary file 1 [file ijms-26-02632-s001.zip › Figure S1.jpg]
